# Editorial: Stress-induced flowering in plants

**DOI:** 10.3389/fpls.2023.1338150

**Published:** 2023-11-30

**Authors:** Dongliang Qiu, Yuri Shavrukov, Jianjun Chen, Li-Yu Chen

**Affiliations:** ^1^ College of Horticulture, Fujian Agriculture and Forestry University, Fuzhou, China; ^2^ College of Science and Engineering (Biological Sciences), Flinders University, Adelaide, SA, Australia; ^3^ Mid-Florida Research and Education Center, Environmental Horticulture Department, University of Florida, Apopka, FL, United States; ^4^ Engineering Research Center for Sugarcane, Fujian Agriculture and Forestry University, Fuzhou, China

**Keywords:** plants, stress-induced flowering, metabolism, gene expression, signaling, regulation mechanism

Global climate change is inducing a rise in the occurrence and intensity of abiotic stresses on plants. Flowering is a sensitive stage of ontogenesis. Flowering plants have evolved mechanisms to modulate the time to flower and flowering duration in order to secure seed production. It is known that flowering is regulated by photoperiod and/or vernalization. Flowering can also be affected by stress. A variety of stress variables are closely related to climate changes, such as temperature and drought as well as other variables including light, nutrients, oxygen, and pruning, which can induce or accelerate flowering, or inhibit or delay flowering.

Molecular mechanisms underlying stress-induced flowering are complex. High temperatures in winter usually delay flowering, such as the active expression of repressors like *FLOWERING LOCUS C* (*FLC*) in *Arabidopsis* and *VERNALIZATION2* (*VRN2*) in winter wheat. In contrast, higher temperatures in spring and summer can accelerate flowering in spring-sown species. The expression of *FLOWERING LOCUS T* (*FT*) promoted the interaction with PHYTOCHROME-INTERACTING FACTOR4 (PIF4), resulting in the early onset of flowering. Additionally, methylation or demethylation of DNA, and changes in histones can accelerate or slow-down the time to flower.

The objective of this Research Topic was to update the progress being made on the understanding of stress-induced flowering. A total of six articles were accepted into this Research Topic, which covered cassava, tobacco, litchi, tomato, foxtail millet, and snapdragon. A group of 52 authors contributed to this Research Topic.

Cassava (*Manihotesculenta* Crantz) is a popular root crop providing carbohydrates to more than 800 million people, but how to induce desirable parental plants to flower earlier for breeding purposes has been a challenge. Rodrmguez et al evaluated photoperiod, pruning, and growth regulator application for flowering induction. Photoperiod extension reduced the time to flower from 6-7 months to 3-4 months for those late-flowering parental plants. Seed production increased using pruning and plant growth regulator 6-benzyladenine. The combination of photoperiod extension, pruning, and plant growth regulators not only induced early flowering but also significantly increased fruit and seed production. Through this research, the authors developed ‘a flower-inducing technology’ for effectively facilitating new cultivar development of cassava.

Cold environmental conditions often facilitate plants to flower. Mechanisms regulating cold induced flowering in tobacco (*Nicotiana tabacum* L.) were studied by Dai et al. Transcriptomic analysis identified a series of differentially expressed genes implicated in cold induced early flowering. Further analysis showed that genes related to brassinosteroid (BR) biosynthetic pathway, circadian system, and flowering pathway were significantly upregulated by cold treatment. As a result, there was an increased accumulation of brassinolide and decreased expression of *FLC*. The BR signaling gene, *NtBIR1* played a critical role in early flowering induction.

Litch (*Litchi chinensis* Sonn.) is an important fruit crop tree in southern China and other subtropical regions. Litch flowering is mainly induced by cold temperature, but irregular flowering time has substantially affected fruit production. Shan et al studied the involvement of CBF in the fine-tuning of litchi flowering time. The cold treatment decreased the expression of *LcCBF1*, *LcCBF2*, and *LcCBF3*, but binding *LcCBF2* and *LcCBF3* to the promoter of *LcMFT* activated their expression. Ectopic overexpression of *LcCBF2* and *LcCBF3* in *Arabidopsis* resulted in delayed flowering, and overexpression of *LcMFT* in *Arabidopsis* had no significant effect on flowering time. The authors believed that prolonged low temperatures were likely to suppress the expression of *LcCBF2* and *LcCBF3*, thus inducing flowering in litchi crops.

High ambient temperatures affect flowering time and also subsequent floral organ development, fertilization, and fruit development. The fruit setting of tomato (*Solanum lycopersicon* L.) is known to be reduced by high temperatures due to the reduction in pollen viability. Cui et al investigated if a fungal extract from an endophytic *Paecilomyces variotii*, called Zhinengcong (ZNC), could decrease high temperature effects in tomato. Results showed that application of ZNC alleviated heat stress by downregulating the expression of genes involved in the production of reactive oxygen species (ROS) and upregulating genes in antioxidant production, thus preventing the accumulation of heat-induced ROS in anthers, pollen grains, and pollen tubes. Foliar spraying of ZNC promoted fruit setting and crop yield of tomato.

Foxtail millet (*Setariaitalica* P. Beauvois) is a small grain crop, and ambient temperatures regulate heading date (HD) or flowering time. To elucidate the molecular basis of HD, Huang et al studied the expression of 14 key flowering time (FT) genes in four accessions at different ambient temperatures. The authors found that the expression levels of *SiEhd1*, *SiFT11*, and *SiCO4* were positively correlated, but *SiPRR95*, *SiPRR1*, *SiPRR59*, *SiGhd7-2*, *SiPHYB*, and *SiGhd7* expressions were negatively correlated with ambient temperatures. Moreover, the expression levels of *SiGhd7-2*, *SiEhd1*, *SiFT*, and *SiFT11* were significantly associated with HD. This study resulted in the establishment of a co-expression regulatory network, which may serve as a foundation for breeding foxtail millet cultivars in response to global warming.

Snapdragon (*Antirrhinum majus* L.) is a model plant used for investigating flower development. In the report of Han et al., molecular mechanisms involved in blue light induced floral terpenoid biosynthesis was studied. The authors found that the exposure of snapdragon flowers to blue light activated blue light signal key receptor AmCRY1. The light signal was then transduced to transcription factor AmMYB24 through interaction with AmCRY1, and final AmMYB24 activated AmOCS by binding to its MYBCOREATCYCBE motif, resulting in the release of abundant ocimene. This research showed the complex regulation of terpenoid production in response to blue light exposure in snapdragon flowers.

## Final remarks

Publications within this Research Topic showcase recent advancements in understanding stress-induced flowering. Evidently, stress-induced flowering is a multifaceted process intricately tied to the genetic composition of plant species and their dynamic interplay with environmental elements. The response to the same stressors can vary significantly among different plant species. Notably, the molecular-level response to cold temperatures differed markedly between tobacco and litchi. This Research Topic has no papers on drought, nutrition, oxygen stress induced flowering. Thus, further research on this subject is anticipated.

## Author contributions

DQ: Writing – original draft, Writing – review & editing. YS: Writing – original draft, Writing – review & editing. JC: Writing – original draft, Writing – review & editing. LC: Writing – review & editing.

